# Psychometric evaluation of the ethical caring competency scale among Iranian Nurses: A methodological study

**DOI:** 10.1371/journal.pone.0346672

**Published:** 2026-05-08

**Authors:** Yasin Jafari Rahim Abad, Kiyana Saadati, Fatemeh Ghaffari

**Affiliations:** 1 Student Research Committee, Nursing Care Research Center, Health Research Institute, Babol University of Medical Sciences, Babol, Iran; 2 Student Research Committee, Ramsar Campus, Mazandaran University of Medical Sciences, Ramsar, Iran; 3 Nursing Care Research Center, Health Research Institute, Babol University of Medical Sciences, Babol, Iran; 4 Department of Nursing, Faculty of Medical Sciences, Tarbiat Modares University, Tehran, Iran; South China Normal University, CHINA

## Abstract

**Background:**

Nurses face complex ethical challenges across diverse healthcare settings, making ethical caring competency essential to professional practice. Given that Iranian nurses encounter ethically sensitive situations shaped by sociocultural and religious values and that no validated tool exists to assess this construct the aim of this study was to culturally adapt and psychometrically validate the Ethical Caring Competency Scale (ECCS) for use among Iranian nurses.

**Methods:**

This methodological, cross‑sectional study included 697 registered nurses who were recruited through convenience sampling from seven teaching hospitals affiliated with Mazandaran University of Medical Sciences, Iran. Data were collected in person by the researcher through visits to hospital wards, during which nurses completed the instruments at the end of their work shifts. The Ethical Caring Competency Scale (ECCS; 22 items across four dimensions) was culturally adapted for the Iranian context following World Health Organization (WHO) translation protocols, including forward–back translation, expert panel review, and pilot testing with 20 nurses. Construct validity was evaluated using Exploratory Factor Analysis (EFA), Confirmatory Factor Analysis (CFA), Exploratory Graph Analysis (EGA), and the Random Forest Model (RFM). In addition, internal consistency reliability and measurement invariance across gender were examined.

**Results:**

All 697 nurses fully completed the data collection instruments. Participants had a mean age of 35.17 ± 8.41 years and 10.55 ± 7.24 years of work experience; most were female (80.6%) and held a bachelor’s degree (89.4%). EFA identified a four‑factor structure that explained 52.80% of the total variance. CFA results confirmed an adequate model fit, with all fit indices (PCFI = 0.737, PNFI = 0.713, CMIN/DF = 2.76, RMSEA = 0.071, IFI = 0.934, CFI = 0.933, and GFI = 0.918) meeting acceptable criteria. EGA results also reproduced the four-factor structure in 99.4% of bootstrap samples. Convergent validity (AVE > 0.5) and divergent validity (√AVE > inter-construct correlations) were confirmed. All reliability coefficients were above 0.7. The RFM demonstrated that the component ‘Acting while thinking about how to provide better care’ was the most significant factor (contributing 37.56%) in predicting the total ethical caring competency score. The measurement invariance test indicated that the factor structure was stable across genders.

**Conclusions:**

The Persian version of the ECCS demonstrated acceptable psychometric properties, a stable conceptual structure, cultural sensitivity, and good discriminant validity for the components of ethical caring competency. Therefore, it can be considered a valid and reliable instrument for assessing the ethical caring competency of nurses in Iran.

## Background

Ethical caring competency is a core and essential capability in the nursing profession [1]. It refers to nurses’ integrated ability to recognize ethical issues, reason morally, and act in accordance with professional and humanistic values to provide morally appropriate care [2,3]. Nurses continually encounter complex situations that necessitate rapid and sound decision-making. These situations can include ethical conflicts related to maintaining patient confidentiality, upholding justice in care delivery, and resolving disputes within the healthcare team [2]. Nurses’ ethical caring competency in managing these ethical situations and making appropriate decisions is key to enhancing the quality of care, fostering patient trust, and reducing clinical errors [4]. Conversely, deficiencies in ethical caring competency can lead to serious consequences in clinical settings, such as increased ethical conflicts, diminished quality of care, a rise in patient complaints, and decreased job satisfaction among nurses [5]. Nurses who lack sufficient ethical caring competency may experience burnout and moral distress when faced with challenging situations. This, in turn, contributes to an increased likelihood of turnover among nurses, which can lead to nursing staff shortages, heavier workloads for remaining nurses, and a decline in the overall quality and continuity of patient care  [6–9]. For this reason, healthcare and educational organizations emphasize the development of ethical caring competency through continuous training and ethical supervision [10,11].

In the Iranian healthcare system, nurses frequently encounter ethically complex situations that are distinctly shaped by the country’s sociocultural and religious context. For example, decisions regarding end‑of‑life care, truth‑telling to patients (especially in cases of poor prognosis), and family‑centered decision‑making often involve navigating deeply held religious beliefs (e.g., Islamic principles on sanctity of life, divine will) and strong familial hierarchies. Moreover, issues such as gender‑segregated care, modesty considerations during physical examinations, and the expectation of providing care within the framework of ‘ta’arof’ (Iranian ritual politeness) can create unique ethical dilemmas that are less prominent in Western or secular healthcare settings. These context‑specific challenges necessitate a culturally sensitive ethical competency that goes beyond universal professional codes. Therefore, developing and maintaining ethical caring competency is not only a professional requirement but also a moral expectation within Iran’s culturally grounded nursing practice [12–16].

Furthermore, to achieve ethically-grounded care, health systems require nurses who can make sound ethical decisions in critical situations while concurrently upholding professional ethical principles, including preserving human dignity, ensuring justice in service provision, and advocating for patient rights [1,4]. Studies indicate that enhancing these competencies through educational programs contributes to reducing ethical conflicts and increasing nurses’ resilience in high-stress environments [17]. Therefore, the regular assessment of ethical caring competency and ensuring its presence among nurses is considered a crucial step in guaranteeing the quality of healthcare [18].

Despite the utility of these instruments, most of them concentrate on only one or a few dimensions of ethical caring competency [11]. For example, the Moral Competence Scale for Home Care Nurses (MCSHCN) primarily assesses ethical analysis and decision‑making in home‑care settings, but does not systematically evaluate dimensions such as “creating indirect effects to provide better care” or “acting to learn what better care is.” [19]. Similarly, the Moral Competence Scale (MCS) focuses on ethical values within a specific cultural context (Thai culture) and does not cover the full spectrum of ethical caring behaviors [20]. The Care Ethical Competence Scale (CECS) is tailored for nursing students and emphasizes ethical attitudes and sensitivity, yet it lacks items that measure the actual performance of ethical care in clinical practice [21]. Likewise, the Moral Competence Questionnaire for Public Health Nurses (MCQ‑PHN) provides a comprehensive assessment for public‑health nurses but is not designed to capture the day‑to‑day ethical challenges faced by hospital‑based nurses across diverse clinical departments [22]. Consequently, these tools, while valuable for their specific purposes, do not offer a holistic measurement of the four core dimensions of ethical caring competency as defined by the Ethical Caring Competency Scale (ECCS). The aim of ECCS is to assess nurses’ ability in ethical decision-making and performance in caregiving situations. The comprehensiveness of this instrument has led to the ECCS being accepted as a standard tool in Japan. Psychometric results indicate that this instrument possesses high validity and reliability and can be effectively used in clinical settings for the comprehensive assessment of nurses’ ethical caring competency [6].

Given that nursing in Iran operates within a unique moral and cultural context strongly influenced by religious and social values [23], the direct use of Western‑ or East‑Asian‑developed tools may not fully capture local ethical perspectives. Therefore, a culturally grounded psychometric validation of the ECCS is essential to ensure conceptual equivalence, cultural relevance, and measurement accuracy among Iranian nurses [24]. Accordingly, this study sought to address the following research question: What are the psychometric properties including validity and reliability of the Persian version of the ECCS among Iranian nurses?

## Objectives

This study aimed to culturally adapt and psychometrically validate the ECCS for use among Iranian nurses

## Materials and methods

### Study design

This study employed a methodological, cross-sectional design aimed at the cultural adaptation and psychometric validation of the ECCS. The cross-sectional design was chosen because it allowed assessment of the factor structure, validity, and reliability of the instrument within a single time frame, without any intervention. Participant recruitment was conducted from 20 July 2024–15 January 2025. The study population comprised all registered nurses working in seven hospitals located in the western region of Mazandaran Province and affiliated with Mazandaran University of Medical Sciences, Iran. These hospitals were selected based on their geographical accessibility and representativeness of major clinical environments, including Intensive Care Unit (ICU), Cardiac Care Unit (CCU), Post-CCU, Internal Medicine, Surgery, Emergency, and Oncology wards. This study was part of a larger project (contract code 724135862) at the Health Research Institute, Babol University of Medical Sciences.

### Population and sampling

The inclusion criteria were as follows: (a) being officially employed as a registered clinical nurse in one of the hospitals affiliated with Mazandaran University of Medical Sciences located in the western part of the province; (b) having at least one year of clinical work experience; and C willingness to participate and provide informed written consent. Exclusion criteria included failure to complete the instruments fully or withdrawing from the study before submission. The sample size was determined based on the rule of thumb that there should be at least 10 participants for each scale item, i.e., an ideal ratio of respondents to items is 10:1 [25]. Considering 22 items in the ECCS instrument and allowing for possible attrition, a total of 697 nurses were included. In addition to this rule, the final sample size was set to approximately 697 to account for the elimination of incomplete responses, the 20‑participant pilot test, and the need to ensure adequate statistical power for advanced analyses including Exploratory Graph Analysis (EGA), Random Forest Model (RFM), and Measurement Invariance testing, as well as to allow for split‑sample validation (EFA n = 348 and CFA n = 349). Sampling was conducted using the convenience method.

### Data collection process

The data collection process followed a structured and sequential procedure and was conducted systematically and in person. After obtaining official approval from the Deputy of Research of Mazandaran University of Medical Sciences and the nursing directors of the seven selected teaching hospitals, the first author who was solely responsible for the entire data collection process obtained a list of registered nurses employed in each hospital from the nursing administration offices. No research assistants or data collection personnel were involved, as the researcher independently managed data collection through a pre‑planned schedule across hospitals and work shifts. Accordingly, data were gathered sequentially over a six‑month period, enabling the researcher to visit each hospital in turn and cover all three work shifts (morning, evening, and night). In coordination with nursing managers and head nurses, the researcher entered each ward, introduced herself, explained the study objectives and significance, and invited nurses to voluntarily participate. Nurses who agreed to take part provided written informed consent, and the paper‑based survey instruments were distributed to them in person. To accommodate the shift‑based nature of nursing work and to prevent interference with patient care, the researcher arranged a suitable time with each participant for questionnaire completion and follow‑up. The completed instruments were collected by the researcher personally at the pre‑agreed time in each ward. On average, participants completed all sections within approximately 10–15 minutes. This method ensured that data collection was carried out in an organized, ethical, and interference‑free manner while maintaining confidentiality, voluntary participation, and the accuracy of responses.

Data were collected using the following instruments:

### Demographic characteristics questionnaire

This questionnaire included variables such as age, gender, work experience, work shift, job satisfaction, income adequacy, financial status, educational level, department of service, marital status, and type of employment within the organization.

### Ethical Caring Competency Scale (ECCS)

The ECCS was originally developed by Katayama et al.  (2022) to assess nurses’ ethical caring ability in clinical practice. The instrument comprises 22 items organized into four dimensions: “Expressing the sensitivity and value of good care” (4 items), “Acting while thinking about how to provide better care” (9 items), “Creating indirect effects to provide better care” (6 items), and “Acting to learn what better care is” (3 items). Each item is rated on a five‑point Likert scale ranging from 1 (never) to 5 (always). The total possible score ranges from 22 to 110, with higher scores indicating a greater level of ethical caring competency. Although the original developers did not define a diagnostic cut‑off, the current study used a distribution‑based tertile classification to facilitate interpretation: scores 22–58 reflect low, 59–86 reflect moderate, and 87–110 reflect high ethical caring competency. The internal consistency reliability of the original instrument for its four dimensions ranged from Cronbach’s α = 0.72 to 0.89 [6].

### Translation

The translation and cultural adaptation of the ECCS were carried out following the World Health Organization (WHO) protocol for instrument adaptation. First, the original English version of the instrument was translated into Persian independently by two bilingual translators (Forward Translation). The two forward‑translated versions were then reviewed and synthesized by a multidisciplinary panel comprising translators, nursing researchers, and medical‑ethics experts to produce a single reconciled version (Reconciliation). Next, the reconciled Persian version was translated back into English by two native English translators who were unaware of the original instrument (Back Translation). This step aimed to ensure semantic and conceptual consistency between the source and target versions. Subsequently, an expert committee consisting of researchers, linguists, and ethics specialists compared all versions and finalized the culturally and linguistically appropriate Persian version (Expert Committee Review). Finally, the pre‑final version was tested with a group of 20 nurses from the target population (Pre‑testing) to confirm clarity, comprehensibility, and cultural relevance of the translated items. After this stage, the finalized version of the ECCS was prepared for psychometric evaluation.

## Psychometric evaluation process of ECCS

### Face validity

In this study, the face validity of the instrument was assessed both qualitatively and quantitatively. In the qualitative stage, 12 nurses from the target population reviewed the Persian version of the ECCS and provided comments on the clarity, simplicity, and cultural relevancy of the items. According to their feedback, three items (Items 2, 4, and 20) underwent minor rewording in wording and sentence structure to enhance clarity, simplicity, and cultural appropriateness within the Iranian clinical context. After incorporating these adjustments, quantitative face validity was assessed. In this phase, the same 12 nurses rated item importance on a 5‑point Likert scale, and all item impact scores exceeded 1.5, confirming that every item was clear, comprehensible, and highly relevant to the intended construct [26].

### Content validity

The content validity of the instrument was assessed both qualitatively and quantitatively. In the qualitative content validity stage, a group consisting of 12 experts in the fields of nursing, professional ethics, and psychometrics were invited to review the instrument in terms of clarity, appropriateness of the items to the intended concept, and content comprehensiveness. In this stage, items identified as inappropriate by the experts were rewritten to better align with the instrument’s objectives. To assess quantitative content validity, two indices, the Content Validity Ratio (CVR) and the Content Validity Index (CVI), were used. To measure CVR, experts were asked to rate the necessity of each item on three levels: essential, useful but not essential, and not essential. Items with a CVR value equal to or greater than 0.56 were retained as essential items in the instrument [27]. The CVR was calculated using the following formula:


$$\text{CVR strict}=\;\frac{{\text{n}}_{\text{e}}-\text{N}/\text{2}}{\text{N}/\text{2}}$$

Where nₑ represents the number of experts who rated the item as “essential,” and N denotes the total number of experts participating in the content validity assessment.

After determining the necessity of the items, experts rated the Clarity, Simplicity, and Relevance of each item on a 4-point Likert scale, and then the CVI value for each item was calculated. Items with a CVI value equal to or greater than 0.79 are considered to have acceptable content validity [28,29]. Furthermore, the average CVI for the entire instrument (S-CVI/Ave) was also calculated. An S-CVI/Ave value above 0.90 indicates a desirable level of content validity for the entire instrument [29].

### Construct validity

Construct validity was assessed using Exploratory Factor Analysis (EFA) and Confirmatory Factor Analysis (CFA).

### Exploratory factor analysis

EFA was performed on data obtained from a random subsample of 348 participants to examine the underlying factor structure of the 22 items of the ECCS. Data adequacy for factor analysis had been verified prior to extraction. The KMO value should be at least above 0.60 for the data to be considered suitable for factor analysis [30]. Also, Bartlett’s Test of Sphericity was used to examine the significance of the correlation between items. The significance of this test (p < 0.05) indicates the existence of sufficient correlation to perform factor analysis [31,32]. Principal Axis Factoring (PAF) was used for factor extraction, applying the criterion of Eigenvalues > 1. To allow for correlated factors, Promax rotation was performed. The optimal number of factors was determined based on both Scree Plot inspection and Parallel Analysis. Parallel analysis was performed with 1,000 iterations, comparing the real eigenvalues with those obtained from randomly generated datasets of identical size; factors with eigenvalues exceeding the 95th percentile of simulated data were retained. Items with factor loadings below 0.40 were excluded from the model [32].

### Confirmatory factor analysis

CFA was conducted to evaluate the fit of the four‑factor model extracted from EFA using the independent subsample reserved for cross‑validation. Several fit indices were employed to assess the adequacy of model fit, including Chi‑square/df < 3, Comparative Fit Index (CFI) ≥ 0.90, Tucker–Lewis Index (TLI) ≥ 0.90, Root Mean Square Error of Approximation (RMSEA) < 0.08, and Standardized Root Mean Square Residual (SRMR) < 0.08  [33].

### Normal distribution of data, outliers, and missing data

In this study, to improve the accuracy of statistical analyses, the normality of data, outliers, and missing data were examined and managed. The normal distribution of data was assessed using skewness and kurtosis indices (within the ± 1 range) and the Kolmogorov-Smirnov and Shapiro-Wilk tests [34]. To identify outliers, first, univariate outliers were identified using Z-Score ≥ ±3, and then multivariate outliers were identified and removed via Mahalanobis Distance at a significance level of p < 0.001 [32]. Furthermore, missing data were examined for randomness using Little’s test, and if no specific pattern was present, missing data were managed using appropriate methods [35].

### Convergent and discriminant validity

In this study, convergent validity and discriminant validity, as subsets of construct validity, were assessed using CFA and Structural Equation Modeling (SEM). To examine convergent validity, the average variance extracted (AVE) for each construct was calculated. According to the criteria proposed by Fornell & Larcker, an AVE value higher than 0.5 indicates desirable convergent validity and signifies that more than 50 percent of the construct’s variance is explained by its items [36]. Discriminant validity was also assessed based on the Fornell & Larcker method. This method confirms discriminant validity when the square root of the AVE of each construct is greater than its correlation with other constructs. If this condition is met, it indicates that the constructs are well-differentiated from each other, and the instrument has appropriate discriminant validity [36].

### Reliability

The reliability of the ECCS was evaluated through two complementary approaches. Internal consistency was examined using Cronbach’s alpha and McDonald’s omega, which assess the degree to which all items consistently measure the same construct; coefficients above 0.70 were considered acceptable [37,38]. Stability (test–retest reliability) was assessed by re‑administering the scale to a subsample of 30 nurses after a two‑week interval and calculating the Intraclass Correlation Coefficient (ICC) between the two administrations. An ICC greater than 0.70 indicated satisfactory temporal stability of the instrument [39].

### Ceiling and Floor Effects

In this study, the ceiling and floor effects of the ECCS were examined to ensure that the instrument can adequately discriminate among respondents across all levels of the construct. Ceiling and floor effects occur when a considerable proportion of participants achieve the highest or lowest possible scores, which undermines the sensitivity of the instrument. To assess these effects, the score distribution was inspected, and the percentages of respondents obtaining the minimum and maximum scores were calculated. Following the quality criteria proposed by Terwee et al. (2007) a widely accepted guideline for evaluating measurement properties of health status questionnaires a ceiling or floor effect was considered present if more than 15% of respondents reached the extreme scores [40].

### Standard Error of Measurement (SEM)

In this study, SEM was calculated to assess the precision of the instrument and the extent of potential variation in participants’ true scores due to random errors. SEM is a measure that indicates how much an observed score might differ from an individual’s true score (Rejas et al., 2008). For this purpose, the instrument’s reliability coefficient (Cronbach’s alpha) and the standard deviation of the total scores were calculated, and using the relevant formula, the SEM value was obtained. Subsequently, the 95% confidence interval for the true score was estimated using the SEM value for each individual [37,41].

### Feasibility and acceptability

In this study, to assess the feasibility and acceptability of the instrument, several key indicators were examined [37,41]. Feasibility was evaluated through criteria such as the time taken to complete ECCS, data completeness, and clarity of items. To determine the completion time, participants were asked to record the time required to complete the instrument. Also, the percentage of instruments that were fully completed was recorded as an indicator for examining data completeness. Furthermore, in the pilot stage, participants were asked to provide their opinions on the clarity and comprehensibility of the items, and items that were deemed ambiguous or difficult by them were revised. To assess acceptability, indicators including response rate, qualitative feedback from participants, and dropout rate were examined. The percentage of individuals who responded to all items of the instrument was considered as a measure of response rate, and the dropout rate was considered as another indicator for acceptability. Additionally, qualitative feedback was collected from participants regarding their experience of completing ECCS to determine their satisfaction with the instrument.

### Measurement Invariance across Gender

In this research, to assess the consistency of the instrument across gender groups, multi-group analysis based on SEM was used. This approach allows for the examination of an identical factor structure between different groups. In this regard, three levels of invariance, including configural invariance, metric invariance, and scalar invariance, were tested according to the guidelines proposed by Cheung & Rensvold [42]. SEM is a fundamental principle in psychometrics that determines whether a measurement instrument functions in the same way across different groups (such as gender) [43].

### Exploratory Graph Analysis (EGA)

To identify the factor structure of the ECCS, we employed EGA, a novel network‑psychometric approach that represents variables as nodes and their conditional dependencies as edges in a graphical model. After computing the item‑correlation matrix, we used the Graphical LASSO to estimate the partial correlation network, from which the number and composition of factors were derived. This data‑driven, network‑based technique served as a cross‑validation of the factor structure obtained from EFA. The convergence between EFA and EGA results provides empirical evidence for the structural stability of the scale, indicating that the identified dimensions are not sample‑specific but reflect robust latent constructs. For readers less familiar with advanced psychometrics, EGA offers a transparent, visual representation of how items cluster together, thereby enhancing confidence in the scale’s factorial integrity and supporting its practical applicability in diverse nursing contexts [44].

### Random Forest Model (RFM)

RFM was employed as a complementary machine‑learning approach to evaluate the relative importance of each component and item of the ECCS in predicting the total ethical‑caring‑competency score. The model builds an ensemble of decision trees through random sampling of both observations and predictors, which enables it to capture complex, non‑linear relationships among items while effectively preventing overfitting [45]. Beyond its predictive accuracy, the RFM output provides a practical ranking of influential items, thereby identifying which dimensions of ethical caring competency contribute most substantially to the overall score. This information directly supports targeted interventions and assessment prioritization in clinical and educational settings, making the scale not only a measurement tool but also a decision‑aid for nursing managers and educators.

### Statistical analysis

Prior to statistical analyses, data were screened for completeness and accuracy. Incomplete or inconsistent responses (e.g., surveys with more than 20% missing items) were excluded from the final analysis. EFA was performed using SPSS v26 to examine the underlying factor structure of the ECCS. CFA and measurement invariance testing were conducted using the maximum likelihood estimation method in AMOS v24. EGA was implemented using the EGAnet package in R, and RFM analyses were performed using the randomForest package in R to assess item and component importance. Descriptive statistics, skewness and kurtosis evaluation, and ceiling/floor effects were calculated using SPSS. Reliability indices, including Cronbach’s alpha, were estimated via SPSS, and McDonald’s omega was computed using the psych package in R. Convergent and discriminant validity were assessed based on the Fornell and Larcker criterion, with AVE and CR calculated in AMOS v24.

### Ethics approval and consent to participate

The study was conducted in accordance with the Declaration of Helsinki and received ethical approval from the Ethics Committee of Babol University of Medical Sciences (Code: IR.MUBABOL.HRI.REC.1403.047). All methods were carried out in accordance with the relevant guidelines and regulations. The objectives of the ongoing study were explained to all participants and informed written consent was obtained from them. In details, obtaining permission for audio recording, explaining the objectives and methodology to participants, informing participants of their right to withdraw at any stage of the research, adhering to the principle of non-maleficence, maintaining confidentiality and anonymity of participant information, and offering to share the research results with participants if they wished was part of the ethical considerations in the study.

## Results

### Descriptive statistics

In this study, nurses with a mean age of 35.17 ± 8.41 years, ranging from 22 to 57 years, were examined. Also, the mean work experience of nurses was 10.55 ± 7.24 years, with a range of 1–30 years. The results show that most nurses, in terms of gender, 562 (80.60 percent) were female; in terms of marital status, 439 (63 percent) were married; in terms of education level, 623 (89.4 percent) had a bachelor’s degree; in terms of place of service, 195 (28 percent) were in the emergency department; in terms of work shift, 103 (14.8 percent) had a fixed morning shift; in terms of job satisfaction, 374 (53.7 percent) were average; in terms of income adequacy, 470 (67.4 percent) reported less than sufficient; in terms of financial status, 458 (65.7 percent) were average; and in terms of employment type, 369 (52.9 percent) were permanent.

### Face and content validity

The results of the qualitative face validity assessment showed that most items were deemed appropriate in terms of clarity, simplicity, and relevance to the intended concept. However, some items underwent minor revisions due to cultural incongruity or conceptual ambiguity. For instance, minor linguistic simplifications were made to enhance clarity and cultural appropriateness for example, refining the phrase “having a bird’s‑eye view of caring based on laws, rules, and social trends” to a more accessible wording for nurses while preserving its original meaning. In the quantitative face validity, all items had a value higher than 1.5. This indicated that all items were sufficiently important from the respondents’ perspective, and therefore, no items were deleted at this stage. In the qualitative content validity assessment, experts evaluated all items in terms of conceptual appropriateness, clarity, and comprehensiveness. Some items were revised after receiving corrective feedback, but no items were completely deleted. The CVR value for all items was equal to or higher than 0.56, indicating the necessity of all items according to the experts. The CVI value for all items was also equal to or higher than 0.79, indicating confirmation of the instrument’s content validity. The overall S-CVI/Ave mean was calculated to be higher than 0.90, which confirmed the desirable level of the instrument’s content validity.

### Exploratory factor analysis

The results show that the sample size is adequate for performing factor analysis, with a KMO equivalent to 0.948, indicating that the data are sufficient for analysis. Also, Bartlett’s test was significant (${\text{x}}^{\text{2}}=\text{6293}.\text{16},\text{ P}<\text{0}.\text{001}$). Therefore, it indicates that there is sufficient correlation between the items to proceed with factor analysis. The EFA results indicate the presence of four factors in the ECCS, including: Acting while thinking about how to provide better care, creating indirect effects to provide better care, Expressing the sensitivity and value of good care, and acting to learn what better care is. These four latent factors explain 19.56 percent, 13.37 percent, 10.41 percent, and 9.46 percent of the variance, respectively, and in total, they explained 52.80% of the total variance of ECCS. All items had an acceptable factor loading higher than 0.4 (Table 1). The final Persian version of the ECCS retained all the 22 original items and confirmed the same four‑factor structure identified in the original scale. No items were deleted after factor extraction, and the Likert‑type scoring (ranging from 1 = Never to 5 = Always) as well as the total score range (22–110) remained unchanged.

**Table 1:** Extracted Exploratory Factors after Promax Rotation.

Factor	Item No. & Statement	Factor Loading	Communality (h²)	Variance Explained (%)	Eigenvalue
**Acting while thinking about how to provide better care**	5. Evaluation of care is based on reactions such as the words and behavior of the patient and (or) their family.	0.767	0.678	19.56	4.30
	6. Practice good care patiently without giving up.	0.706	0.634		
	7. Introduce evidence into practice with appropriate procedures.	0.816	0.714		
	8. Support the patient and (or) their family to gain essential awareness.	0.641	0.603		
	9. Support decision making in the way and at the pace the patient and (or) their family wants and create a care plan together.	0.664	0.605		
	10. Perspective taking of the patient and (or) their family’s experience by observing behavior and integrating multifaceted information.	0.902	0.717		
	11. Estimate patient’s subjective distress from physical assessment.	0.624	0.551		
	12. Create relationships so the patient and (or) their family can talk about important things.	0.551	0.557		
	13. Accept the patient and (or) their family facing the rigors of reality.	0.439	0.564		
**Creating indirect effects to provide better care**	14. Flexibly create contextual care teams based on professionalism and expertise.	0.571	0.688	13.37	2.94
	15. Create and modify systems for good care, such as conferences.	0.909	0.696		
	16. Understand and discuss the conflicts of team members based on multidisciplinary collaboration.	0.802	0.712		
	17. Disseminate and raise issues without ignoring the challenges of performing good care.	0.757	0.658		
	18. Understand and discuss conflicts between colleagues or department members.	0.535	0.840		
	19. Exchange opinions and participate in conferences in order to carry out good care.	0.536	0.622		
**Expressing the sensitivity and value of good care**	1. Expressing values about good care in individual cases.	0.661	0.579	10.41	2.28
	2. Having a bird’s-eye view of the pros and cons of caring based on laws, rules and social trends.	0.674	0.628		
	3. Exploring diverse values and awareness without sticking to own values.	0.934	0.800		
	4. Feeling conflicted and uncomfortable about situations where good care is not being provided.	0.725	0.546		
**Acting to learn what better care is**	20. Discover and disseminate ethical research issues from practice.	0.711	0.767	9.46	2.08
	21. Learn about good care from reflection and insights based on the experience case.	0.947	0.859		
	22. Gain awareness about good care practices at learning opportunities.	0.824	0.789		

**Fig 1:**
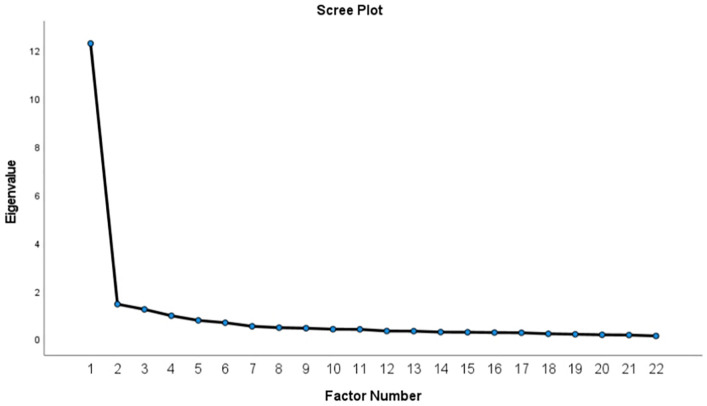
Scree plot based on eigenvalues for factor extraction.

To confirm the four factors in ECCS, a scree plot was also used. As observed in Fig 1, the presence of four factors is also confirmed based on the scree plot.

### Confirmatory factor analysis

To evaluate the model fit, all indices: PCFI = 0.737, PNFI = 0.713, CMIN/DF = 2.76, RMSEA = 0.071, IFI = 0.934, CFI = 0.933, and GFI = 0.918 confirmed the appropriate fit of the model. Therefore, the four-factor model of ethical care competency was confirmed (Table 2).

**Table 2:** Fit Indices of the ECCS Confirmatory Factor Analysis Model.

Fit Indices	${𝐱}^{2}$	df	P-value	CMIN/df	RMSEA (CL90%)	PNFI	CFI	PCFI	IFI	GFI
First-Order	558.07	202	<0.001	2.76	0.071 (0.068- 0.075)	0.713	0.933	0.737	0.934	0.918
Second-Order	587.20	204	<0.001	2.87	0.073 (0.070- 0.076)	0.716	0.929	0.741	0.929	0.912

**Fig 2:**
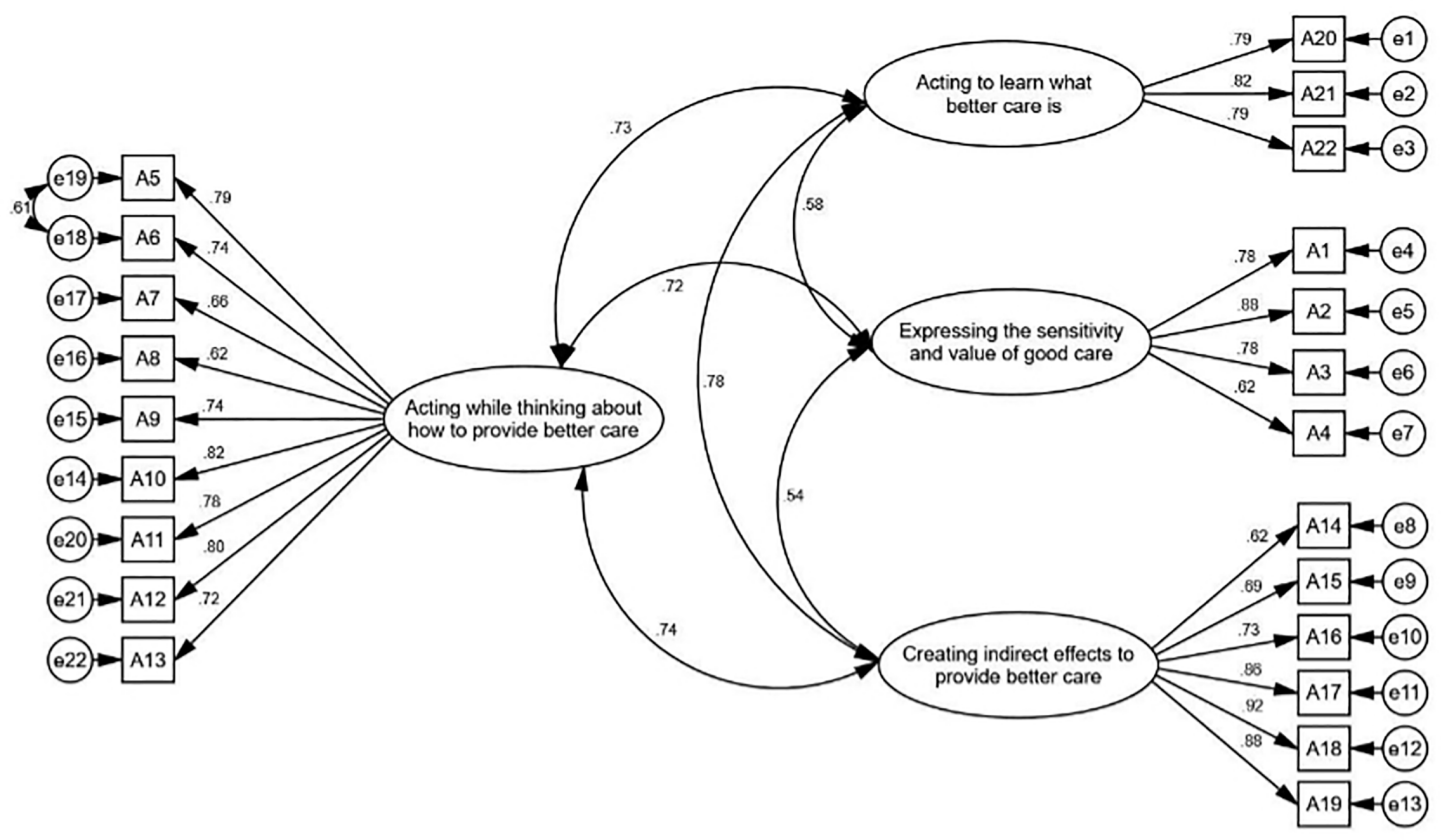
First-Order Confirmatory Factor Analysis of ECCS.

Fig 2 shows the standardized factor loadings between the items and the factors of the ethical care competency construct in the first-order factor analysis. All factor loadings in the model are above 0.4.

**Fig 3:**
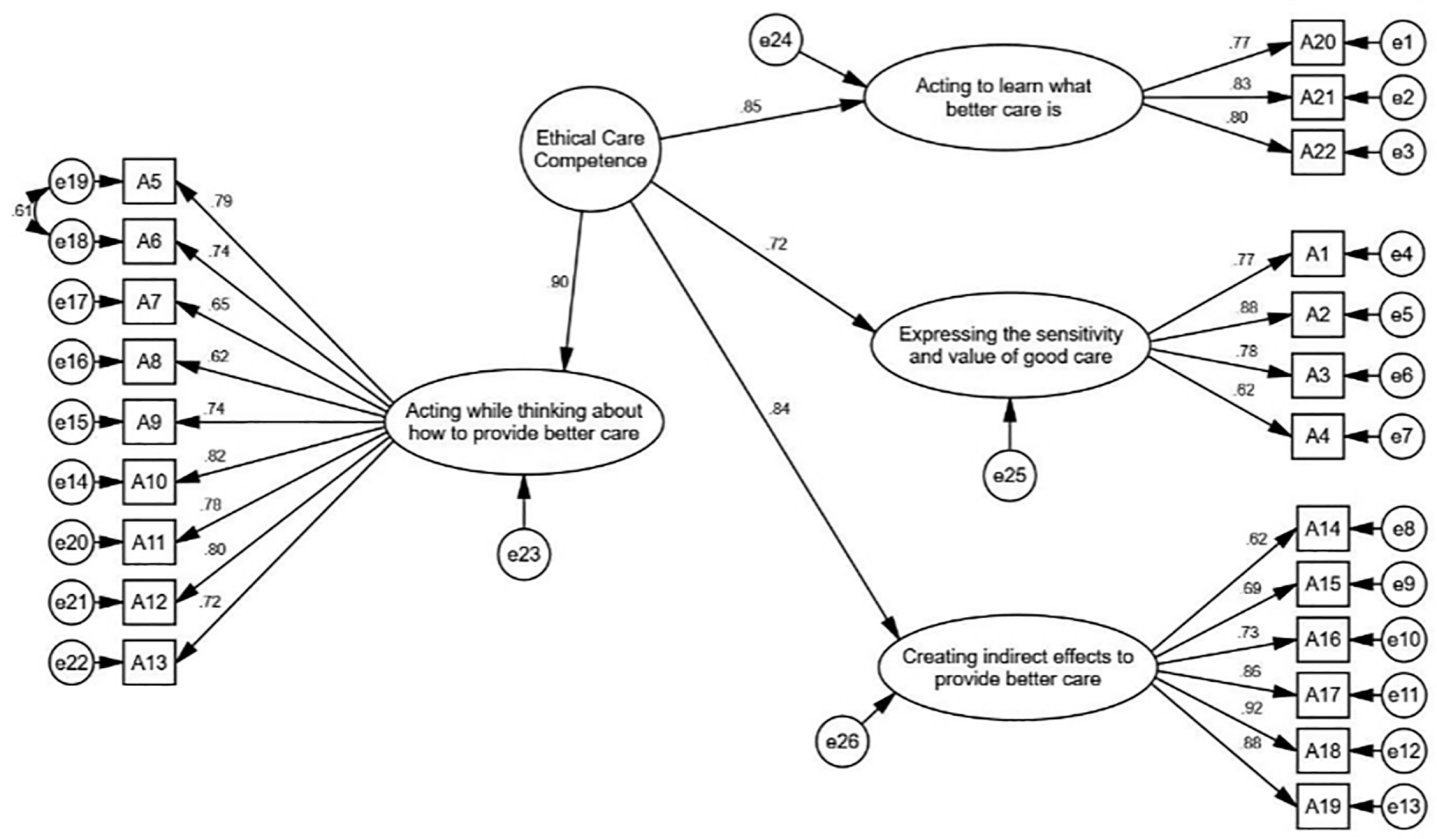
Second-Order Confirmatory Factor Analysis of ECCS.

Fig 3 shows the standardized factor loadings of each factor and the construct in the second-order confirmatory factor analysis. In the second-order confirmatory factor analysis, the values of the fit indices indicate an acceptable fit of the proposed model with the data.

### Convergent and discriminant validity

The results obtained from calculating AVE showed that all constructs have values higher than 0.5, and therefore, the convergent validity of the instrument was confirmed. Also, comparing the square root of AVE for each construct with its correlation with other constructs showed that the condition proposed by Fornell and Larcker holds for all constructs. These findings indicate that the instrument has desirable discriminant validity, and the measured constructs are well-differentiated from each other (Table 3).

**Table 3:** Convergent Validity, Discriminant Validity, Internal Consistency, and Stability of ECCS Factors.

Factor	α	Ω	CR	AVE	ICC
Acting while thinking about how to provide better care	0.926	0.926	0.933	0.553	0.833
Creating indirect effects to provide better care	0.911	0.909	0.908	0.626	0.842
Expressing the sensitivity and value of good care	0.853	0.854	0.852	0.594	0.857
Acting to learn what better care is	0.878	0.878	0.842	0.640	0.890
Total	0.957	0.956	0.898	0.689	0.934

### Reliability

The findings related to Reliability showed that ECCS has very good internal consistency and temporal stability. Cronbach’s alpha (α) and McDonald’s omega (Ω) coefficients for all factors were reported to be higher than 0.85, indicating high consistency among the items of each factor. Also, CR was greater than 0.84 for all factors, indicating the desirable structural stability of the instrument. Furthermore, ICC values are in the range of 0.83 to 0.89, which signifies high stability in repeated assessments (Table 3).

### Ceiling and floor effects assessment

In this study, the ceiling and floor effects for the four factors are less than 15%; therefore, no ceiling and floor effects are observed, and the total score is normally distributed (Table 4).

**Table 4:** Ceiling and Floor Effects.

Factor	Ceiling Effect	Floor Effect	Skewness	Kurtosis
Acting while thinking about how to provide better care	0%	6.5%	0.17	0.15
Creating indirect effects to provide better care	0.1%	7.5%	0.33	0.22
Expressing the sensitivity and value of good care	0%	9.5%	0.18	0.24
Acting to learn what better care is	0%	14.1%	0.32	0.15
Total	0%	4%	0.10	0.08

### Standard Error of Measurement (SEM)

The examination of SEM in the ethical competency construct shows that the instrument used has appropriate precision for evaluating different components. SEM values in all subscales are relatively low, ranging from 0.70 to 2.14, which indicates acceptable measurement error. Also, the Minimum Detectable Change (MDC) values in all components are greater than SEM, and the Minimum Important Change (MIC) values are also lower than MDC in all cases, which indicates the instrument’s ability to identify meaningful and clinical changes. The presence of positive agreement in all components also strengthens the construct validity of the results (Table 5).

**Table 5:** Standard Error of the Ethical Competency Construct.

Factor	Mean (Standard Deviation)	Min-Max	SEM	MDC	MIC	Agreement
Acting while thinking about how to provide better care	18.60 (5.24)	9-43	2.14	5.94	0.37	Positive
Creating indirect effects to provide better care	13.24 (4.12)	6-30	1.63	4.54	0.22	Positive
Expressing the sensitivity and value of good care	8.13 (2.27)	4-16	0.85	2.38	0.16	Positive
Acting to learn what better care is	6.36 (2.10)	3-14	0.70	1.94	0.09	Positive
Total	46.34 (12.08)	22-100	3.10	8.60	0.72	Positive

### Feasibility and acceptability

The findings showed that ECCS performs adequately in terms of Feasibility and Acceptability. The average time required to complete the scale was 10 minutes. 96.4% of the scales were fully completed, indicating data completeness. In the pilot phase, only 3.2% of participants reported some items as ambiguous, and these items were revised after review. In terms of acceptability, 94.8% of participants responded to all items of the instrument, and the dropout rate throughout the instrument completion process was 1.7%. Also, 89% of respondents reported their overall experience of completing the instrument as “satisfactory” or “completely satisfactory.” These results indicate that the ECCS instrument not only has conceptual accuracy but is also simple to implement, understandable, and acceptable to the target population.

### Measurement invariance across gender

The results of the multi-group confirmatory factor analysis (MGCFA) showed that the 4-factor model of ECCS has a good fit in both male and female groups. In the first stage, by imposing the constraint of equality of factor loadings between the two groups, no significant difference in the $x^{\text{2}}$value was observed ($\Delta {\text{x}}^{\text{2}}(\text{18})=\text{24}.\text{49},\text{ p}=\text{0}.\text{139}$), which indicates the establishment of factor loading invariance. Subsequently, by constraining variances and factor covariances, the results still indicated a good fit of the model and no significant difference ($\Delta {\text{x}}^{\text{2}}(\text{28})=\text{32}.\text{33},\text{ p}=\text{0}.\text{261}$), which indicates structural invariance. In the third step, by also imposing constraints on the measurement residuals, the model still had a good fit ($\Delta {\text{x}}^{\text{2}}(\text{51})=\text{63}.\text{12},\text{ p}=\text{0}.\text{118}$). Also, the ΔCFI index in all stages of analysis (for factor loadings = 0.001, for covariances = 0.002, and for residuals = 0.005) was less than the threshold of 0.01, which means maintaining model invariance at a desirable level. However, although invariance in the factor structure and relationships between factors was established in the male and female groups, at the level of measurement residuals, signs of difference were observed, which may be due to cultural or experiential differences between the two groups (Table 6).

**Table 6:** Results of Multi-Group Confirmatory Factor Analysis Designs in Different Subgroups.

Model	${𝐱}^{2}$	df	${𝐱}^{2}/𝐝𝐟$	CFI	GFI	RMSEA	Δ𝐂𝐅𝐈	Δ𝐑𝐌𝐒𝐄𝐀
Multi-group Comparison								
Model without constraints	1732.40	404	4.28	0.907	0.903	0.069		
Model with constraint on factor loadings	1756.89	422	4.16	0.906	0.900	0.067	0.001	0.003
Model with constraint on structural covariances	1764.73	432	4.08	0.905	0.899	0.066	0.002	0.003
Model with constraint on measurement residuals	1795.52	455	3.96	0.902	0.896	0.065	0.005	0.004

### Exploratory Graph Analysis (EGA)

**Fig 4:**
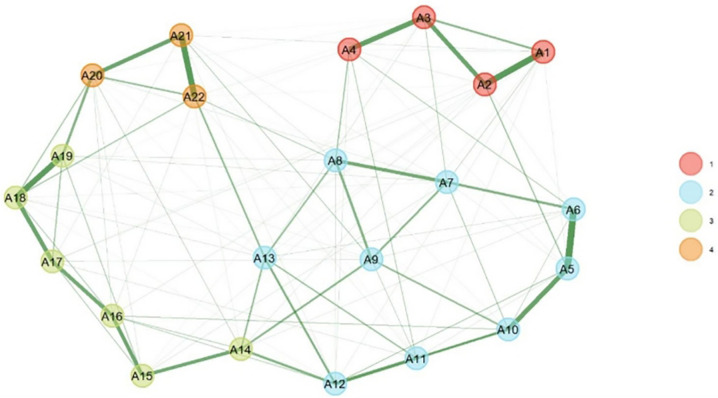
Results of Network Analysis Factors for ECCS.

EGA identified and estimated the four-factor structure of ECCS. The results showed that all items were correctly clustered with their respective factors, and this structure aligns with the findings from EFA. Furthermore, bootstrap analysis with 1000 iterations showed that the median number of factors was four, with a standard error of 0.060. The 92% confidence interval for the number of factors was between 4.19 and 6.47. The results indicated that in 99.4% of the bootstrap samples (994 out of 1000 samples), a four-factor structure was identified. These findings indicate the stability and predominance of the four-factor structure in the data and support the factorial validity of the scale at the network level (Fig 4).

### Random forest model

**Fig 5:**
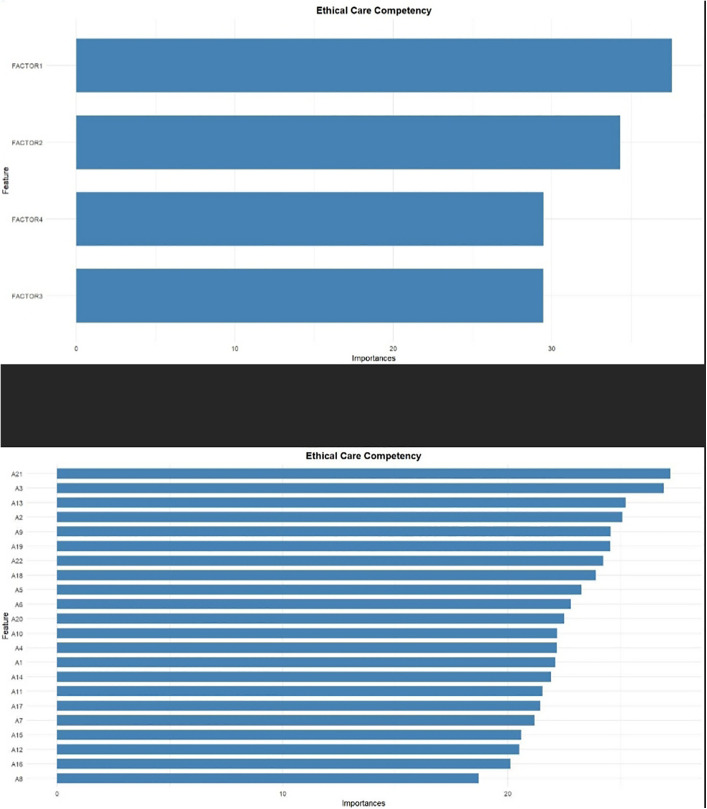
Variable importance plot derived from the Random Forest model for the ECCS.

The results show that the component “acting while thinking about how to provide better care” with 37.56% was the most important component, and the items “learn about good care from reflection and insights based on the experience case” and “exploring diverse values and awareness without sticking to own values” with 27.21% and 26.92% respectively, were identified as the most important items (Fig 5).

## Discussion

The overall aim of this research was to examine the cultural adaptation of ECCS in the Iranian nursing community and to evaluate its psychometric properties. In this regard, the process of translation and cultural adaptation of the scale was carried out based on standard frameworks. One of the key stages in this research was the examination of the factorial structure of the scale using EFA. Before performing EFA, statistical indices were evaluated to check the adequacy of the data. The KMO measure of sampling adequacy was 0.948, which is above the desirable threshold of 0.90 and indicates excellent data adequacy for factor analysis [46]. Also, Bartlett’s test of sphericity was significant (p < 0.001), which implies sufficient correlation between items and the suitability of the data for factor analysis [30,31]. The EFA results showed that ECCS has a suitable factorial structure, and its four main factors, including “acting while thinking about how to provide better care,” “creating indirect effects to provide better care,” “expressing the sensitivity and value of good care,” and “acting to learn what better care is,” were clearly differentiated. These four factors collectively explained 52.80% of the total variance. All items had factor loadings higher than 0.40, and in each factor, one item with the highest factor loading was identified, indicating the stability and appropriate differentiability of the instrument’s dimensions [32]. Also, the results of the study by Katayama et al. (2022) on the Japanese version of the scale confirm the current findings; in that study, the KMO value was reported as 0.931, Bartlett’s test of sphericity was significant, and the four ECCS factors were able to explain 65.3% of the total variance [6]. Although the amount of variance explained by these four factors in the present study is considerably lower compared to the original Japanese version of the instrument, this significant difference could be due to several factors, including cultural and contextual differences in understanding ethical concepts, and heterogeneity in sample characteristics (in terms of education, work experience, or work environment). Although the proportion of explained variance in the Iranian sample was lower than that reported in the Japanese validation, this does not necessarily indicate a weaker construct. In psychometric research, explained variance values above 50% are generally considered acceptable for multidimensional constructs [47,48]. Moreover, the consistency of the four-factor structure suggests that the ECCS captures the core ethical caring competencies relevant to Iranian nursing practice. Hence, the instrument remains applicable within this cultural context, albeit with contextual nuances that may influence the salience of certain ethical behaviors.

From a conceptual perspective, each factor of the ECCS reflects essential dimensions of moral practice in Iranian nursing. The first factor, “acting while thinking about how to provide better care,” emphasizes cognitive–ethical integration in clinical judgment. In the Iranian context, this dimension resonates with nurses’ responsibility to align technical competence with the principles of beneficence and human dignity, particularly when resources are limited or moral distress occurs [8,49]. The second factor, “Creating indirect effects to provide better care,” represents nurses’ ability to foster ethical climates through teamwork and institutional support. Iranian nurses often act as moral mediators among physicians, patients, and families; thus, this domain highlights collaborative ethics and organizational justice within hospital culture [50]. The third factor, “expressing the sensitivity and value of good care,” underscores moral sensitivity and the ability to recognize ethical issues in day‑to‑day patient care. This trait has been strongly associated with enhanced empathy and reduced moral disengagement in Eastern healthcare systems [51]. Finally, “acting to learn what better care is” reflects the dynamic, reflective learning process through which Iranian nurses update moral understanding based on experience and professional self‑evaluation. Reflection‑in‑practice is considered a key strategy to strengthen ethical resilience and self‑awareness [15]. Collectively, these factors illustrate how ECCS not only measures abstract moral reasoning but also captures applied ethical competence shaped by Iran’s cultural and religious context.

The CFA findings in this research confirmed the good fit of the four-factor structure of ECCS among Iranian nurses. Statistical indices, including CFI, IFI, GFI, and RMSEA, were all within acceptable ranges, indicating the alignment of the instrument’s conceptual structure with empirical data [52,53]. In particular, an RMSEA value of 0.071 and a CFI of 0.933 indicate a good fit of the model with the data, and these results are consistent with international standards for confirmatory factor analysis [52]. Furthermore, all factor loadings were reported above 0.40, which is a common criterion for the adequacy of correlation between items and factors in structural equation modeling [54]. Comparing these findings with the original version of the instrument also supports the stability of the factorial structure in the new cultural context. Therefore, it can be concluded that the Farsi version of ECCS possesses a confirmed, stable, and suitable factorial structure for use in nursing ethics research.

The findings from EGA also showed that the four-factor structure of ECCS is reproducible and replicable in the Iranian nursing population. The results indicated that items were correctly clustered within their respective factors, and this clustering was consistent with the EFA results. The stability of this factorial structure was assessed using bootstrap analysis, and in 99.4% of the resampled instances, the four-factor structure was maintained. This high replicability indicates the stability of the instrument’s factorial structure at different sampling levels [55]. The median number of factors in the bootstrap samples indicates high precision in estimating the number of factors. These results clearly demonstrate that EGA, as a novel method complementary to traditional analyses, can be very effective in evaluating the conceptual structure of psychometric scales, especially in diverse cultural contexts.

In the present study, the RFM findings showed that the component “Acting while thinking about how to provide better care,” with a contribution of 37.56%, was the most important dimension in predicting the total score of the nursing ethical care competency construct. This dimension represents the nurse’s ability to continuously evaluate professional actions, analyze care situations, and apply ethical judgment in practice; characteristics that form the foundation of valid ethical competency [56]. The two items, “learn about good care from reflection and insights based on the experience case” and “Exploring diverse values and awareness without sticking to own values,” which had the highest importance, are also fully aligned with this construct. These items indicate that nurses with reflective skills and flexibility in their value systems can better face complex ethical situations and make ethically-sound decisions. In the conceptual literature, ethical care competency is also defined as a combination of knowledge, attitude, and skill for analyzing and responding to ethical situations in clinical care [1,56,57].Therefore, the present findings strengthen the validity of this construct, as the dimension identified through RFM precisely covers the operational components of this definition. These results emphasize the importance of ECCS, which measures not only overt ethical behaviors but also the capacity for reflection, value analysis, and professional learning as integral parts of ethical competency in nursing.

In this study, the results of the measurement invariance analysis confirmed that ECCS can be used for comparisons between men and women without structural bias. The present study, through the analysis of measurement invariance, has gone a step further than the research by Katayama et al. and has been able to provide greater validity to the ECCS instrument for inter-group comparisons based on gender. This enhances the generalizability of the scale in future studies and its clinical application in diverse nursing communities. Furthermore, to further enhance the scale’s comparability across different nursing subpopulations, future research could examine measurement invariance across other key grouping variables, such as years of work experience or clinical departments [42,43].

### Strength and limitations

This study is considered one of the most comprehensive research projects conducted on the cultural adaptation and evaluation of the psychometric properties of ECCS in the Iranian nursing community. To ensure the accuracy, validity, and cultural compatibility of the instrument, standard cross-cultural adaptation frameworks, including forward and backward translation, review by a panel of experts, and pilot testing in the target population, were utilized. This research examined all psychometric dimensions, including face, content, and construct validity (through EFA, CFA, and EGA), as well as convergent and divergent validity. Furthermore, the Feasibility and Acceptability of the instrument were assessed, and its reliability was evaluated from two aspects: Cronbach’s alpha and McDonald’s omega for internal consistency, and through test-retest for assessing the temporal stability of the instrument.

Among the prominent innovations of this study was the use of EGA as a novel method for examining the factorial structure, which confirmed the high stability of the ECCS four-factor structure. Also, the examination of measurement invariance based on gender showed that the factorial structure of the instrument is stable between male and female nurses and allows for inter-group score comparisons. Moreover, in this research, RFM was used for the first time to analyze the relative importance of the factors and items of the ECCS instrument; an approach not employed in the Japanese version of the scale. This analysis enabled the precise identification of the most effective components influencing ethical care competency and introduced a novel approach to the psychometric content analysis of ethical instruments. The use of a large and diverse sample (n = 697) from various hospital departments has also increased the generalizability of the findings to the Iranian nursing community.

Despite its numerous strengths, this research is also accompanied by limitations. The cross-sectional design, while appropriate for psychometric validation, precludes causal inference and does not allow examination of the temporal stability of ethical caring competency. The use of a convenience sampling method may reduce the generalizability of the results to the entire population of nurses. Furthermore, the research’s focus on nurses working in hospitals in a specific geographical region (west of Mazandaran province) could subject the findings to the influence of regional cultural and organizational factors. However, it should be noted that Iran’s healthcare delivery system is highly centralized; all public hospitals nationwide operate under standardized policies and professional protocols mandated by the Ministry of Health and Medical Education. Therefore, organizational culture and professional practices are largely similar across provinces, minimizing potential regional bias. In addition, because the vast majority of Iranian nurses share a common religious background, where respect for patients and protection of patients’ rights are emphasized as fundamental moral and religious duties, the ethical orientation underlying nursing practice is relatively homogeneous across the country. This cultural and religious consistency may further reduce regional variation in ethical caring behaviors. It is recommended that in future studies, this instrument be evaluated in more geographically and professionally diverse populations. Future longitudinal or repeated-measure designs would be valuable to examine changes in ethical caring competency over time and to establish causal relationships.

### Clinical implications

The results of this study confirmed that ECCS, due to its ability to identify weaknesses in nurses’ ethical competency, can be used in in-service training, monitoring nurses’ performance, and as a basis for developing professional improvement programs. Hospital managers and nursing managers can use ECCS to assess the level of ethical competency of nurses and utilize its results in designing managerial strategies to promote ethical standards in healthcare settings. Furthermore, this instrument is applicable in developing specialized educational programs in the field of professional ethics. Conducting skill-enhancement courses and training workshops based on ECCS data plays an important role in improving ethical decision-making processes and strengthening nurses’ professional skills. On the other hand, the self-assessment capability provided by ECCS allows nurses to independently gauge their ethical competencies, identify their strengths and weaknesses, and, based on that, develop individual improvement strategies. This process helps increase ethical self-awareness and enhance the quality of decision-making in complex clinical situations. Finally, the widespread use of ECCS can provide valuable information to health policymakers and contribute to the development of guidelines based on professional ethics. At a macro level, this will lead to the improvement of care standards, enhancement of the quality of nursing services, and strengthening of ethical principles in the healthcare system.

## Conclusion

The present research was conducted with the aim of validating and examining the psychometric properties of ECCS in the Iranian nursing community. The findings showed that this instrument possesses desirable validity and reliability, as well as measurement invariance across different groups. The comprehensive approach of this research in examining various aspects of the instrument’s validity and reliability has helped clarify the factorial structure of ECCS and reinforces its potential application in future research and professional development programs for nurses.

## Abbreviations

ECCS: Ethical Caring Competency Scale
α: Cronbach’s alpha coefficients
Ω: McDonald omega coefficient
CR: Construct Reliability
AVE: Average Variance Extracted
ICC: Intraclass Correlation Coefficient
CFA: Confirmatory Factor Analysis
CMIN/DF: Chi-square/degree-of-freedom ratio
RMSEA: Root Mean Square Error of Approximation
PCFI: Parsimonious Comparative Fit Index
GFI: Goodness of Fit Index
PNFI: Parsimonious Normed Fit Index
IFI: Incremental Fit Index
CFI: Comparative Fit Index
CVR: Content Validity Ratio
CVI: Content Validity Index
WHO: World Health Organization
EGA: Exploratory Graph Analysis
RFM: Random Forest Model
PAF: Principal Axis Factoring.

## References

[1] Lechasseur K, Caux C, Dollé S, Legault A. Ethical competence: An integrative review. Nurs Ethics. 2018;25(6):694–706. doi: 10.1177/0969733016667773 27694548

[2] Kulju K, Stolt M, Suhonen R, Leino-Kilpi H. Ethical competence: A concept analysis. Nurs Ethics. 2016;23(4):401–12. doi: 10.1177/0969733014567025 25670176

[3] Numminen O, van der Arend A, Leino-Kilpi H. Nurses’ codes of ethics in practice and education: a review of the literature. Scand J Caring Sci. 2009;23(2):380–94. doi: 10.1111/j.1471-6712.2008.00608.x 19077064

[4] Robichaux C. Ethical competence in nursing practice: competencies, skills, decision-making. Springer Publishing Company. 2016.

[5] Numminen O, Repo H, Leino-Kilpi H. Moral courage in nursing: A concept analysis. Nurs Ethics. 2017;24(8):878–91. doi: 10.1177/0969733016634155 27005953

[6] Katayama H, Muramatsu T, Aoki Y, Nagashima E. Psychometric evaluation of the Ethical Caring Competency Scale in nursing. BMC Nurs. 2022;21(1):103. doi: 10.1186/s12912-022-00886-2 35505338 PMC9066827

[7] Poikkeus T, Numminen O, Suhonen R, Leino-Kilpi H. A mixed-method systematic review: support for ethical competence of nurses. J Adv Nurs. 2014;70(2):256–71. doi: 10.1111/jan.12213 23865484

[8] Borhani F, Abbaszadeh A, Nakhaee N, Roshanzadeh M. The relationship between moral distress, professional stress, and intent to stay in the nursing profession. J Med Ethics Hist Med. 2014;7:3. 25512824 PMC4263391

[9] Ashghaly Farahani M, Oskouie F, Ghaffari F. Factors affecting nurse turnover in Iran: A qualitative study. Med J Islam Repub Iran. 2016;30:356. 27453886 PMC4934483

[10] Falkenström E, Ohlsson J, Höglund AT. Developing ethical competence in healthcare management. Journal of Workplace Learning. 2016;28(1):17–32. doi: 10.1108/jwl-04-2015-0033

[11] Koskenvuori J, Stolt M, Suhonen R, Leino-Kilpi H. Healthcare professionals’ ethical competence: A scoping review. Nurs Open. 2018;6(1):5–17. doi: 10.1002/nop2.173 30534390 PMC6279725

[12] Joolaee S, Nikbakht-Nasrabadi A, Parsa-Yekta Z, Tschudin V, Mansouri I. An Iranian perspective on patients’ rights. Nurs Ethics. 2006;13(5):488–502. doi: 10.1191/0969733006nej895oa 16961113

[13] Zafarnia N, Abbaszadeh A, Borhani F, Ebadi A, Nakhaee N. Moral competency: meta-competence of nursing care. Electron Physician. 2017;9(6):4553–62. doi: 10.19082/4553 28848630 PMC5557135

[14] Mobasher M, Aramesh K, Zahedi F, Nakhaee N, Tahmasebi M, Larijani B. End-of-life care ethical decision-making: Shiite scholars’ views. J Med Ethics Hist Med. 2014;7:2. 25512823 PMC4263386

[15] Ajoudani F, Baghaei R, Lotfi M. Moral distress and burnout in Iranian nurses: The mediating effect of workplace bullying. Nurs Ethics. 2019;26(6):1834–47. doi: 10.1177/0969733018779210 29938574

[16] Davari Ardakani N, Pournorouz M, Bagheri F, Kalantari M. Ta ‘arof in Persian Language and Culture: Situations, Strategies and Linguistic Structures (a guideline preparation for compiling TPFL Textbooks). Journal of Sociolinguistics. 2024;7(1):45–67.

[17] Schaefer R, Vieira M. Ethical competence as a coping resource for moral distress in nursing. Texto contexto - enferm. 2015;24(2):563–73. doi: 10.1590/0104-07072015001032014

[18] Maluwa VM, Maluwa AO, Mwalabu G, Msiska G. Assessment of ethical competence among clinical nurses in health facilities. Nurs Ethics. 2022;29(1):181–93. doi: 10.1177/09697330211010259 34346258

[19] Asahara K, Ono W, Kobayashi M, Omori J, Todome H. Development and psychometric evaluation of the Moral Competence Scale for Home Care Nurses in Japan. J Nurs Meas. 2013;21(1):43–54. doi: 10.1891/1061-3749.21.1.43 23786133

[20] Jormsri P, Kunaviktikul W, Chaowalit A, Ketefian S. Development of Moral Competence Scale (MCS) in nursing practice in Thailand. Thai Journal of Nursing Research. 2004;8:144–58.

[21] Yoshioka E, Kaneko S. Development of the Care Ethical Competence Scale for Nursing Students. J Jpn Acad Nurs Sci. 2019;39(0):316–25. doi: 10.5630/jans.39.316

[22] Asahara K, Kobayashi M, Ono W. Moral competence questionnaire for public health nurses in Japan: scale development and psychometric validation. Jpn J Nurs Sci. 2015;12(1):18–26. doi: 10.1111/jjns.12044 25171177

[23] Memarian R, Salsali M, Vanaki Z, Ahmadi F, Hajizadeh E. Professional ethics as an important factor in clinical competency in nursing. Nurs Ethics. 2007;14(2):203–14. doi: 10.1177/0969733007073715 17425149

[24] Organization WH. Process of translation and adaptation of instruments. http://www.who.int/substance_abuse/research_tools/translation/en/ 2009.

[25] Nunnally J, Bernstein I. Psychometric Theory. 3rd ed. New York: MacGraw-Hill. 1994.

[26] Polit DF, Beck CT. Nursing research: Generating and assessing evidence for nursing practice. Lippincott Williams & Wilkins. 2008.

[27] Lawshe CH. A Quantitative approach to content validity1. Personnel Psychology. 1975;28(4):563–75. doi: 10.1111/j.1744-6570.1975.tb01393.x

[28] Lynn MR. Determination and quantification of content validity. Nurs Res. 1986;35(6):382–5. doi: 10.1097/00006199-198611000-00017 3640358

[29] Polit DF, Beck CT. The content validity index: are you sure you know what’s being reported? Critique and recommendations. Res Nurs Health. 2006;29(5):489–97. doi: 10.1002/nur.20147 16977646

[30] Hill BD. The sequential Kaiser-Meyer-Olkin procedure as an alternative for determining the number of factors in common-factor analysis: A Monte Carlo simulation. Oklahoma State University. 2011.

[31] Black WC, Babin BJ, Anderson RE: **Multivariate data analysis: A global perspective**: Pearson; 2010.

[32] Tabachnick B, Fidell L, Ullman JB. Using multivariate statistics. Boston: Pearson. 2013.

[33] Brown TA. Confirmatory factor analysis for applied research. Guilford Publications. 2015.

[34] Byrne BM. Structural equation modeling with Mplus: Basic concepts, applications, and programming. Routledge. 2013.

[35] Little RJA. A Test of Missing Completely at Random for Multivariate Data with Missing Values. Journal of the American Statistical Association. 1988;83(404):1198–202. doi: 10.1080/01621459.1988.10478722

[36] Fornell C, Larcker DF. Evaluating Structural Equation Models with Unobservable Variables and Measurement Error. Journal of Marketing Research. 1981;18(1):39. doi: 10.2307/3151312

[37] DeVellis RF, Thorpe CT. Scale development: Theory and applications. Sage Publications. 2021.

[38] Dunn TJ, Baguley T, Brunsden V. From alpha to omega: a practical solution to the pervasive problem of internal consistency estimation. Br J Psychol. 2014;105(3):399–412. doi: 10.1111/bjop.12046 24844115

[39] Koo TK, Li MY. A Guideline of Selecting and Reporting Intraclass Correlation Coefficients for Reliability Research. J Chiropr Med. 2016;15(2):155–63. doi: 10.1016/j.jcm.2016.02.012 27330520 PMC4913118

[40] Terwee CB, Bot SDM, de Boer MR, van der Windt DAWM, Knol DL, Dekker J, et al. Quality criteria were proposed for measurement properties of health status questionnaires. J Clin Epidemiol. 2007;60(1):34–42. doi: 10.1016/j.jclinepi.2006.03.012 17161752

[41] Streiner DL, Norman GR, Cairney J. Health measurement scales: a practical guide to their development and use. Oxford University Press. 2024.

[42] Cheung GW, Rensvold RB. Evaluating Goodness-of-Fit Indexes for Testing Measurement Invariance. Structural Equation Modeling: A Multidisciplinary Journal. 2002;9(2):233–55. doi: 10.1207/s15328007sem0902_5

[43] Meredith W. Measurement Invariance, Factor Analysis and Factorial Invariance. Psychometrika. 1993;58(4):525–43. doi: 10.1007/bf02294825

[44] Christensen AP, Golino H. Exploratory Graph Model. 2024.

[45] Louppe G, Wehenkel L, Sutera A, Geurts P. Understanding variable importances in forests of randomized trees. Advances in neural information processing systems. 2013;26.

[46] Kaiser HF. An Index of Factorial Simplicity. Psychometrika. 1974;39(1):31–6. doi: 10.1007/bf02291575

[47] Hair JF, Black WC, Babin BJ, Anderson RE. Multivariate data analysis. In. New Jersey: Pearson Education Inc. 2010.

[48] Henson RK, Roberts JK. Use of exploratory factor analysis in published research: Common errors and some comment on improved practice. Educational and Psychological Measurement. 2006;66(3):393–416.

[49] Nazari AM, Bakhtiari-Dovvombaygi H, Borhani F, Abbaszadeh A, Gholami M, Bakhshalizadeh Rashti S. The relationship between ethical sensitivity, caring behavior and quality of care in nurses: A systematic review. BMC Nurs. 2025;24(1):329. doi: 10.1186/s12912-025-02916-1 40140794 PMC11948935

[50] Sharifikia I, Khoshnood Z, Hosseinnejad A, Farokhzadian J, Rohani C. Exploring a guide for codes of ethics for the development of ethical competence in Iranian nursing students: a systematic review and meta-synthesis. BMC Nurs. 2024;23(1):519. doi: 10.1186/s12912-024-02208-0 39080746 PMC11289922

[51] Alamdari MP, Raiesdana N, Nobahar M, Yavari MB. Comparison of the correlation between moral sensitivity and clinical competence in emergency and intensive care nurses: A cross-sectional-correlation study. Int Emerg Nurs. 2024;75:101483. doi: 10.1016/j.ienj.2024.101483 38936275

[52] Hu L, Bentler PM. Cutoff criteria for fit indexes in covariance structure analysis: Conventional criteria versus new alternatives. Structural Equation Modeling: A Multidisciplinary Journal. 1999;6(1):1–55. doi: 10.1080/10705519909540118

[53] Schreiber JB, Nora A, Stage FK, Barlow EA, King J. Reporting Structural Equation Modeling and Confirmatory Factor Analysis Results: A Review. The Journal of Educational Research. 2006;99(6):323–38. doi: 10.3200/joer.99.6.323-338

[54] Hair JF, Black WC, Babin BJ, Anderson RE. Multivariate data analysis. Cengage Learning EMEA. 2019.

[55] Golino HF, Epskamp S. Exploratory graph analysis: A new approach for estimating the number of dimensions in psychological research. PLoS One. 2017;12(6):e0174035. doi: 10.1371/journal.pone.0174035 28594839 PMC5465941

[56] Yoshioka E, Kaneko S. Concept Analysis of Ethical Competence of Nursing Students and Nurses. OJN. 2019;09(11):1173–87. doi: 10.4236/ojn.2019.911086

[57] Zia T, Sabeghi H, Mahmoudirad G. Problem-based learning versus reflective practice on nursing students’ moral sensitivity. BMC Nurs. 2023;22(1):215. doi: 10.1186/s12912-023-01377-8 37340373 PMC10283247

